# Effect of L-Arginine on Titin Expression in Rat Soleus Muscle After Hindlimb Unloading

**DOI:** 10.3389/fphys.2019.01221

**Published:** 2019-09-20

**Authors:** Anna Ulanova, Yuliya Gritsyna, Nikolai Salmov, Yuliya Lomonosova, Svetlana Belova, Tatyana Nemirovskaya, Boris Shenkman, Ivan Vikhlyantsev

**Affiliations:** ^1^Institute of Theoretical and Experimental Biophysics, Russian Academy of Sciences, Pushchino, Russia; ^2^Pushchino State Institute of Natural Sciences, Pushchino, Russia; ^3^State Scientific Center RF, Institute of Biomedical Problems, Russian Academy of Sciences, Moscow, Russia

**Keywords:** L-arginine, titin, muscle atrophy, nitric oxide, skeletal muscle

## Abstract

Nitric oxide (NO), produced by NO-synthases via L-arginine oxidation, is an essential trigger for signaling processes involved in structural and metabolic changes in muscle fibers. Recently, it was shown that L-arginine administration prevented the decrease in levels of the muscle cytoskeletal proteins, desmin and dystrophin, in rat soleus muscle after 14 days of hindlimb unloading. Therefore, in this study, we investigated the effect of L-arginine administration on the degree of atrophy changes in the rat soleus muscles under unloading conditions, and on the content, gene expression, and phosphorylation level of titin, the giant protein of striated muscles, able to form a third type of myofilaments—elastic filaments. A 7-day gravitational unloading [hindlimb suspension (HS) group] resulted in a decrease in the soleus weight:body weight ratio (by 31.8%, *p* < 0.05), indicating muscle atrophy development. The content of intact titin (T1) decreased (by 22.4%, *p* < 0.05) and the content of proteolytic fragments of titin (T2) increased (by 66.7%, *p* < 0.05) in the soleus muscle of HS rats, compared to control rats. The titin gene expression and phosphorylation level of titin between these two groups were not significantly different. L-Arginine administration under 7-day gravitational unloading decreased the degree of atrophy changes and also prevented the decrease in levels of T1 in the soleus muscle as compared to HS group. Furthermore, L-arginine administration under unloading resulted in increased titin mRNA level (by 76%, *p* < 0.05) and decreased phosphorylation level of T2 (by 28%, *p* < 0.05), compared to those in the HS group. These results suggest that administration of L-arginine, the NO precursor, under unloading decreased the degree of atrophy changes, increased gene expression of titin and prevented the decrease in levels of T1 in the rat soleus muscle. The results can be used to search for approaches to reduce the development of negative changes caused by gravitational unloading in the muscle.

## Introduction

Nitric oxide (NO) is an essential trigger for signaling processes that lead to structural and metabolic changes in muscle fibers (Shenkman et al., 2015b). It is produced by NO-synthases during oxidation of the amino acid L-arginine. NO-synthase actively participates in the regulation of protein and energy metabolism in skeletal muscles, including the regulation of protein synthesis and degradation (Shenkman et al., 2015b).

Neuronal isoform (nNOSμ), associated with dystrophin in the subsarcolemmal region, is mostly expressed in skeletal muscles (Nakane et al., 1993; Brenman et al., 1995). Neuronal NO-synthase is regulated by intracellular Ca^2+^ via Ca^2+^-dependent binding of the enzyme to the calcium-calmodulin complex (Forstermann et al., 1991). Neuronal NO-synthase is also activated by its substrate L-arginine, which is metabolized to NO (Shenkman et al., 2015b).

Muscle NO concentration has been shown to increase with an increase in muscle contractile activity (Perez et al., 2002; Vassilakopoulos et al., 2003; Pye et al., 2007) and decrease after muscle unloading (Lomonosova et al., 2011). Unloading leads to skeletal muscle atrophy accompanied with excessive degradation of the giant sarcomeric proteins, titin and nebulin (Shenkman et al., 2002; Udaka et al., 2008; Ulanova et al., 2015). The calcium-dependent cysteine proteases, μ-calpain (calpain-1) and m-calpain (calpain-2), have been reported to be involved in the initiation of proteolysis of titin and nebulin (Goll et al., 2003; Shenkman and Nemirovskaya, 2008; Mohrhauser et al., 2011).

It was found that NO inhibited m-calpain activity *in vitro* via S-nitrosylation of the active site cysteine (Koh and Tidball, 2000). It was also shown that NO produced endogenously by the skeletal muscles and other cell types has the potential to inhibit m-calpain activity and cytoskeletal proteolysis (Koh and Tidball, 2000; Samengo et al., 2012). Moreover, age-related loss of NO-synthase in the skeletal muscles was reported to decrease calpain S-nitrosylation, thereby leading to increased myofibril degradation and sarcopenia (Samengo et al., 2012). In addition, the proteolytic susceptibility of titin, the giant elastic protein present in vertebrate striated muscles (Tskhovrebova and Trinick, 2017; Herzog, 2018; Kellermayer et al., 2018; Linke, 2018), has been reported to be affected by titin phosphorylation (Gritsyna et al., 2017).

Nitric oxide is also known to be involved in the regulation of gene expression; brain-derived neurotrophic factor was reported to trigger NO synthesis and S-nitrosylation of histone deacetylase 2 in neurons, resulting in changes to histone modifications and gene activation (Nott et al., 2008).

Changes in titin gene expression were observed in mice skeletal muscles that were atrophied after a 30-day-long space flight (Ulanova et al., 2015). Increased titin degradation and reduced titin content were also observed. Studies of changes in the expression of the titin-coding gene (TTN) in conditions of simulated gravitational unloading have not been conducted. Recently, it was shown that L-arginine administration decreased the degree of atrophy changes and prevented the decrease in levels of the muscle cytoskeletal proteins, desmin and dystrophin, in rat soleus muscle during unloading (Lomonosova et al., 2011). However, the precise mechanism underlying the prevention of muscle atrophy by L-arginine is not yet known, and the effect of L-arginine on titin gene expression and degradation is not clear. Therefore, in this study, we investigated the effect of L-arginine administration on the (i) degree of atrophy changes in rat soleus muscles after hindlimb unloading, (ii) level and gene expression of titin, and (iii) changes in levels of titin phosphorylation in rat soleus during unloading. New data were obtained showing that administration of L-arginine, the NO precursor, under gravitational unloading decreased the degree of atrophy changes, increased gene expression of titin and prevented the decrease in levels of T1 in the rat soleus muscle.

## Materials and Methods

### Animals and Ethical Approval

Fifteen 3-month-old male Wistar rats (190 ± 5 g) were obtained from the certified Nursery for laboratory animals, Institute of Bioorganic Chemistry of the Russian Academy of Sciences (Pushchino, Moscow region). The animals were housed in a temperature-controlled room under a 12:12 h light–dark cycle; food pellets and water were provided *ad libitum*. This study was carried out in accordance with the recommendations of Grundy (2015) and the European Convention for the protection of Vertebrate Animals used for Experimental and Scientific purposes (Council of Europe number 123, Strasbourg, 1986). The protocol was approved by the Biomedicine Ethics Committee of the Institute of Biomedical Problems of the Russian Academy of Sciences/Physiology section of the Russian Bioethics Committee (protocol no. 421 of April 14, 2016). All efforts were made to minimize the animal pain and suffering. Prior to all surgical procedures, the animals were anesthetized with an intraperitoneal injection of tribromoethanol (240 mg kg^–1^). The depth of anesthesia was evaluated by testing the pedal withdrawal reflex (toe and foot pad pinch).

### Hindlimb Unloading and L-Arginine Administration

The hindlimbs were unloaded using a standard rodent hindlimb suspension (HS)/unloading model (Morey-Holton and Globus, 2002). Briefly, a strip of adhesive tape was applied to the animal’s tail, which was suspended by passing the tape through a swivel attached to a metal bar on the top of the cage. This setup allowed the forelimbs to be in contact with the grid floor and allowed the animals to move around the cage for free access to food and water. The suspension height was adjusted to prevent the hindlimbs from touching any supporting surface while maintaining a suspension angle of approximately 30°. The animals were randomly divided into three groups (*n* = 5/group): (1) C, cage control; (2) HS, 7-day hindlimb suspension; and (3) HSL, 7-day hindlimb suspension + L-arginine. 7 days were chosen because preliminary studies demonstrated the development of atrophy in human soleus fibers and significant decrease in the titin content after 7 days gravitational unloading (Shenkman et al., 2004). Animals from the HSL group were daily administered 500 mg kg^–1^ L-arginine (“NOW Foods”, United States) via intramuscular injections. The dose of L-arginine was previously tested and found to be optimal (Lomonosova et al., 2011). The rats from the C and HS groups were administered an equivalent dose of saline. Under anesthesia, the soleus muscles from the control and unloaded rats were surgically excised from both the hindlimbs, frozen in liquid nitrogen, and stored at –80°C until further analysis.

### RNA Isolation

Total RNA was isolated from the samples using the Aurum^TM^ Total RNA Fatty and Fibrous Tissue kit, according to the manufacturer’s protocol (Bio-Rad Laboratories, Inc., Hercules, CA, United States). The total RNA quality was assessed by visualizing the integrity of the 18S and 28S rRNA after electrophoresis in 1% agarose gels. Total RNA concentration was determined via spectrophotometry (NanoDrop ND-1000 spectrophotometer; Nano-Drop Technologies, Waltham, MA, United States). RNA solutions were stored at −75°C.

### Reverse Transcription-Quantitative Polymerase Chain Reaction

Isolated mRNA was reverse-transcribed to cDNA using the M-MLV reverse transcriptase (Evrogen, Moscow, Russia) and oligo-dT_15_ primers (catalog number SB001, Evrogen). Primers specific for gene fragments of titin and GAPDH were selected using Vector NTI software. The following primer pairs were used: titin (N2A isoform) 5′-CAGCAGCCAAGAAAGCCGCT-3′ (forward), 5′-CACCACTCTGATACTCTGAGGCTCTG-3′ (reverse) and GAPDH gene 5′-GCAAGAGAGAGGCCCTCAG-3′ (forward), 5′-TGTGAGGGAGATGCTCAGTG-3′ (reverse). Nucleotide gene sequences were obtained from the NCBI GenBank database. Real-time PCR was performed using a DT-322 amplifier (DNA-Technology, Moscow, Russia), Taq-DNA polymerase (Evrogen), and SYBR Green I fluorescent dye (Invitrogen, Carlsbad, CA, United States). PCR was performed as follows: (i) hot start at 95°C for 5 min, (ii) denaturation at 95°C for 15 s, (iii) primer annealing at 64°C for 20 s, and (iv) extension at 72°C for 20 s. Stages 2 to 4 were repeated 35 times. The amplification products were analyzed by electrophoresis in a 7% polyacrylamide gel. The PCR products were isolated from the gel according to the Cleanup Standard protocol (Evrogen, Russia). DNA fragments were sequenced in Evrogen. BLAST program software was used for identification of the PCR product (see Supplementary Materials). The changes in titin gene expression were determined according to the 2^–ΔΔct^ method (Livak and Schmittgen, 2001), and the housekeeping gene GAPDH was used for normalization.

### SDS-PAGE Analysis of Titin

Changes in titin-1 (T1, intact titin) and T2 (proteolytic fragments) content were determined using polyacrylamide slab gels (2.2%, 10 × 10 × 0.1 cm). The gels were strengthened with agarose according to the Tatsumi–Hattori technique (Tatsumi and Hattori, 1995) with some modifications reported previously (Vikhlyantsev and Podlubnaya, 2017). Muscle tissues were homogenized in lysis buffer (12 mM Tris HCl, 1.2% SDS, 10% glycerol, 2% β-mercaptoethanol or 75 mM DTT, 5 μg/ml leupeptin, and E64, pH 6.8 to 7.0). To prevent titin degradation at high temperatures (Granzier and Wang, 1993), the samples were incubated for 30 to 40 min at 40°C instead of boiling them (Vikhlyantsev and Podlubnaya, 2017). Protein concentrations were measured using the NanoDrop ND-1000 Spectrophotometer. To ensure that equal protein amounts were loaded into the gels, samples from the control and experimental groups were all run on the same gel. SDS-PAGE analysis was performed using the Helicon VE–10 system (Moscow, Russia) at 8 mA. The gels were stained with Coomassie brilliant blue G-250 and R-250 mixed in a 1:1 ratio.

### Determination of Titin Phosphorylation Levels

The level of titin phosphorylation was determined using a previously described method (Borbély et al., 2009) with minor modifications. The native level of protein phosphorylation was estimated in the gels using the fluorescent dye Pro-Q Diamond (Invitrogen). The gels were incubated in an aqueous solution of 50% ethanol and 10% acetic acid for 12–18 h, washed with distilled water for 30 min, and stained for 1.5 h. The gels were then rinsed in Pro-Q Diamond phosphoprotein gel destaining solution (Invitrogen), and the protein bands containing phosphate groups were visualized using the ChemiDoc^TM^ Touch Imaging System (Bio-Rad). The data were processed using the Image Lab Software (Bio-Rad). Finally, the gels were stained with Coomassie brilliant blue G-250 and R-250, mixed in a 1:1 ratio, to determine the total protein content.

### Densitometry and Statistical Analysis

Gels were digitized, and the data were processed using the TotalLab v1.11 software (Newcastle Upon Tyne, England). To determine the titin content relative to that of the myosin heavy chain, the total optical density of the myosin heavy chain peak and the total optical density of the T1 and T2 peak were determined on the same gel, as described previously (Cazorla et al., 2000). It is known that a specific titin-to-myosin ratio exists in the A-disk of the sarcomere (6 titin molecules per half of a myosin filament in a sarcomere) (Liversage et al., 2001). This approach for titin content measurement is more accurate than the estimation based on the total protein content in the sample. The values are presented as M ± SD, where M is the mean value and SD is the standard deviation. The statistical analysis of the results obtained was carried out with SigmaPlot 11.0 software (Systat Software, Inc., 2008). Since the distribution of some data samples was not normal (Shapiro–Wilk test), we estimated the significance of differences using non-parametric single-factor dispersion analysis for repeated measurements (Kruskal–Wallis One Way Analysis of Variance on Ranks) with the following pairwise comparison by the Tukey test (see Supplementary Materials). The differences were considered statistically significant at *p* < 0.05.

## Results

### Analysis of Atrophic Changes in Rat Soleus Muscle After Hindlimb Suspension

Decreased soleus muscle weight (by 40.5%, *p* < 0.05) and lower soleus weight:body weight ratio (by 31.8%, *p* < 0.05) was observed in the HS rats, compared to the C rats (Table 1). Compared to the C group, the HSL group showed both not significant decline in the soleus muscle weight (by 35.5%, *p* = 0.073), and tendency to reduction in the soleus muscle weight:body weight ratio (by 29.4%, *p* = 0.086) (Table 1).

**TABLE 1:** Animal weight, weight of m. soleus and soleus muscle weight to body weight ratio.

**Groups**	**Animal body weight, g**	**Weight of m. soleus, g**	**m. soleus weight/animal weight, mg/g**
“Control”, *n* = 5	248.1 ± 14.3	0.121 ± 0.007	0.487 ± 0.006
“HS”, *n* = 5	216.7 ± 5.8^∗^	0.072 ± 0.007^∗^	0.332 ± 0.025^∗^
“HSL”, *n* = 5	225.8 ± 13.1	0.078 ± 0.007	0.344 ± 0.010

*^∗^*P* < 0.05 as compared to control group.*

### Titin Content in Rat Soleus Muscle

**FIGURE 1:**
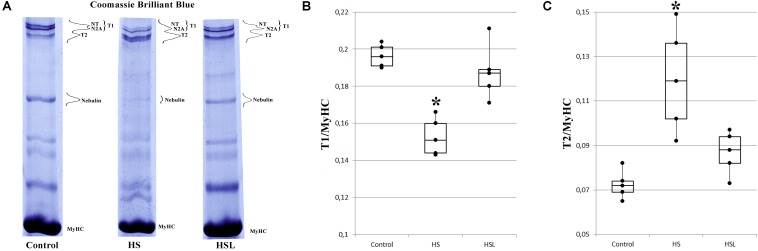
Levels of intact titin-1 (T1) and proteolytic fragments (T2) in the soleus muscle of rats. **(A)** SDS-PAGE analysis of titin expression in the soleus muscle of rats. MyHC – myosin heavy chains. T2 (m.w. ∼2000–2100 kDa) are proteolytic fragments of titin. NT and N2A are isoforms of intact titin-1 (T1, m.w. ∼3400–3700 kDa in skeletal muscles). High molecular weight titin isoforms (denoted as NT-titin) were found in striated muscles of mammals (Vikhlyantsev and Podlubnaya, 2017). Plots of densitometric quantification of the T1 content **(B)** and T2 content **(C)** relative to MyHC content (*n* = 5). ^∗^*P* < 0.05 as compared to control group. Values are means ± SD.

Compared to the C group, the HS group showed a 22.4% decrease in T1 content (*p* < 0.05), and a 66.7% increase in T2 content (*p* < 0.05) in the soleus muscle (Figure 1). The difference in the T1 and T2 contents between the C and HSL groups was not statistically significant (Figure 1). Compared to the HS group rats, the HSL group rats showed a tendency to higher T1 content (by 23%, *p* = 0.086) in the soleus muscles.

### Level of Titin Phosphorylation in Rat Soleus Muscle

**FIGURE 2:**
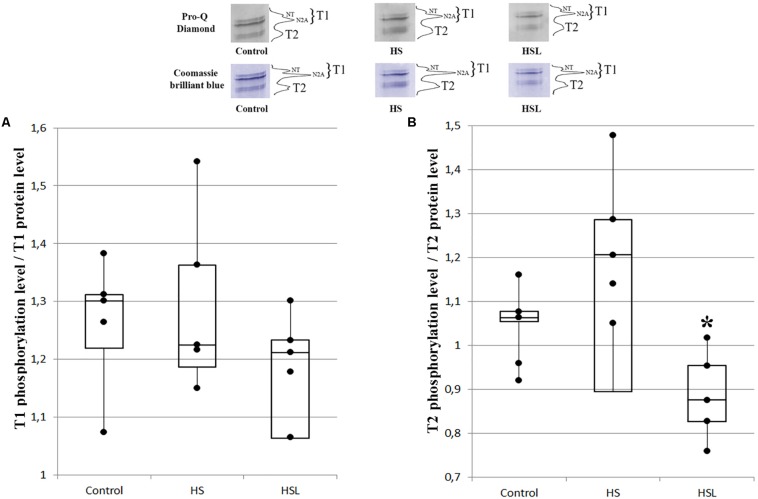
Plots of titin phosphorylation levels in the soleus muscle of rats. **(A)** T1. **(B)** T2. ^∗^*P* < 0.05 when the 7-day hindlimb suspension + L-arginine (HSL) and the 7-day hindlimb suspension (HS) groups were compared, (*n* = 5). Values are means ± SD.

Compared to the C group, the HS group showed a tendency to higher T2 phosphorylation levels (by 18.9%, *p* = 0.333) in the soleus muscles (Figure 2). The level of T2 phosphorylation in the soleus muscle in the HSL group was 14.4% (*p* = 0.234) and 28% (*p* < 0.05) lower than that in the C and HS groups, respectively, Figure 2. The levels of T1 phosphorylation in the soleus muscle in the C, HS, and HSL groups were not significantly different (Figure 2).

### Titin Gene Expression in Rat Soleus Muscle

**FIGURE 3:**
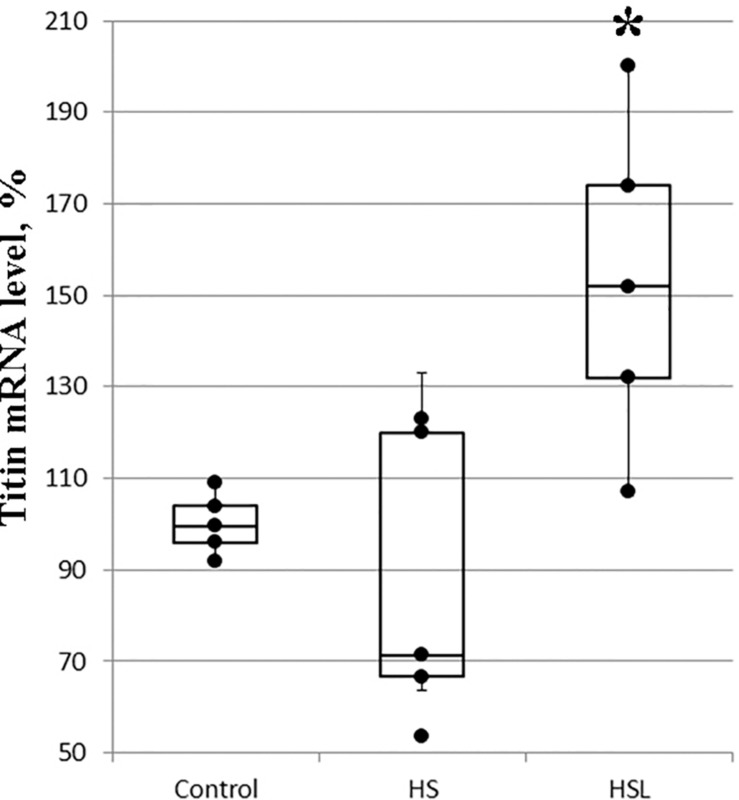
Plots of titin mRNA levels in the soleus muscle of rats (*n* = 5). ^∗^*P* < 0.05 as compared to hindlimb suspension (HS) group. Values are means ± SD.

The differences between the titin mRNA levels on the C and HS groups were not statistically significant (Figure 3), but gene expression analysis revealed a tendency to higher the titin mRNA level (by 53%, *p* = 0.073; Figure 3) in the soleus muscle of rats with L-arginine administration (HSL group), compared to that in rats from the C group. Compared to the HS group, L-arginine administration under 7-day gravitational unloading showed a 76% increase in the titin mRNA level (*p* < 0.05; Figure 3).

## Discussion

### The Effect of L-Arginine Administration on the Degree of Atrophy Changes and Level of Titin in Rat Soleus Muscles After Hindlimb Unloading

It is well known that disuse of muscles under conditions of simulated or real microgravity causes muscle atrophy (Fitts et al., 2001; Baldwin et al., 2013; Hargens and Vico, 2016; Mirzoev and Shenkman, 2018). Our results show a decrease in the weight of soleus muscle relative to the body weight after rat HS, suggesting the development of muscle atrophy. We found that L-arginine decreased the degree of atrophy changes and also prevented the decrease in levels of T1 in rat soleus muscle after 7 days of hindlimb unloading.

Titin is known to be a substrate for calcium-dependent proteases (calpains) (Goll et al., 2003; Huang and Zhu, 2016), and calpain activity is reported to increase in skeletal muscles from the first day of gravitational unloading (Enns et al., 2007). Recent studies have reported that unloading causes rapid skeletal muscle atrophy not only because of increased protein degradation via calpain activation but also because of decreased protein synthesis (Shenkman et al., 2015a; Mirzoev and Shenkman, 2018). Furthermore, it was found that treatment with PD150606 calpain inhibitor prevented the hindlimb suspension-induced atrophy in rat soleus muscle by inhibiting protein degradation pathways and preserving anabolic signaling (Shenkman et al., 2015a). Therefore, it is obvious that calpains play an essential role in the enhanced proteolysis of different proteins, including titin, under gravitational unloading (Enns et al., 2007; Shenkman et al., 2015a). In fact, the development of unloading-induced skeletal muscle atrophy has been shown to be accompanied with a decrease in the content of titin and an increase in the content of its proteolytic fragments T2 (Shenkman et al., 2002, 2004; Toursel et al., 2002; Udaka et al., 2008; Ulanova et al., 2015). Our results are consistent with these reports; compared to the control rats, those subjected to HS showed decreased T1 content and increased T2 content. However, the soleus muscle T1 content remained unaltered after unloading with L-arginine administration, compared to that in the control rats, suggesting a protective effect of L-arginine administration on the protein during unloading. The role of NO as a signaling molecule involved in the regulation of protein metabolism during unloading has recently been discussed (Shenkman et al., 2015b). Moreover, L-arginine administration was shown to prevent the increased proteolysis of desmin and dystrophin in the rat soleus after 14-day gravitational unloading (Lomonosova et al., 2011). In our study the HSL group showed a tendency to decline in the content of T2 proteolytic fragments compared to the HS group. Collectively, our results indicated that NO production is essential for decreasing titin proteolysis under gravitational unloading. These results are consistent with the fact that NO increases calpain S-nitrosylation leading to a decrease in the activity of these proteases (Koh and Tidball, 2000; Samengo et al., 2012).

### The Effect of L-Arginine Administration on the Gene Expression of Titin in Rat Soleus Muscles After Hindlimb Unloading

Changes in titin gene expression and their possible role in alterations of the content of this protein under unloading remains unclear. Titin gene expression was shown to be increased (by 70%) in the atrophied gastrocnemius muscle of mice after a 30-day-long space flight (Ulanova et al., 2015). However, it should be noted that the animals were sacrificed 12 h after they landed; therefore, it was difficult to conclude whether an increase in titin gene expression was a consequence of the effects of microgravity on mice or a result of adaptation to the Earth’s normal environment. We showed that there were no significant changes in titin gene expression in the rat soleus muscle after 7-day simulated gravitational unloading. Therefore, we concluded that alterations in titin gene expression do not play a significant role in unloading-induced changes in the content of this protein.

However, increased titin gene expression in the rat soleus muscle was observed after unloading with L-arginine administration in our work, suggesting the enhanced synthesis of this protein. This increase titin gene expression may be regulated via NO signaling. In 2008, it was shown that brain-derived neurotrophic factor triggers NO synthesis and S-nitrosylation of histone deacetylase 2, subsequently leading to gene activation in neurons (Nott et al., 2008). A later study concluded that cytoskeleton rearrangement and regulation of protein stability and turnover could potentially be modulated through NO-dependent changes in histone deacetylase activity (Watson and Riccio, 2009). NO-dependent machineries are known to be involved in the control of the key protein expression by means of inhibition of sumoylation of most SUMO targets (Qu et al., 2007) or GSK3β inhibition (Drenning et al., 2008; Sharlo et al., 2018). Our data show that NO is involved in the regulation of titin gene expression.

### The Effect of L-Arginine Administration on the Changes in Titin Phosphorylation Levels in Rat Soleus Muscles After Hindlimb Unloading

*In vivo* titin phosphorylation is well known (Somerville and Wang, 1988) and the phosphorylation sites of this protein, located over the length of its molecule in the A-band (T2-titin part), I-band, and Z-disk in the sarcomere, have been determined (Linke and Hamdani, 2014). Many potential phosphorylation sites of titin molecule have also been discovered (Huttlin et al., 2010; Lundby et al., 2012)^1^^,^^2^. Phosphorylation is known to modify the stiffness of titin molecules (Linke and Hamdani, 2014). In recent years, the role of titin phosphorylation in the development of unloading-induced changes in muscles has been discussed. In particular, it was shown that titin hypophosphorylation, reducing the stiffness of titin molecules, may be involved with the development of diaphragm weakness caused by mechanical unloading (Ottenheijm et al., 2011; van Hees et al., 2012; van der Pijl et al., 2019). The role of phosphorylation of titin in change of its susceptibility to proteolysis has also been discussed. Phosphorylation is known to modify the sensitivity of proteins to their degradation by calpain-1 (Di Lisa et al., 1995). For instance, protein kinase A (PKA)-mediated phosphorylation of troponin-I reduced its sensitivity to degradation by calpain-1, whereas phosphorylation of protein kinase C (PKC), contrariwise, increased the sensitivity of troponin-I to proteolysis (Di Lisa et al., 1995). We found no direct experimental evidence to confirm a change in the sensitivity of titin to proteolysis mediated by a change in its phosphorylation level. However, there are indirect data testifying that titin hyperphosphorylation is accompanied by an increase in its proteolytic degradation. Using a fluorescent dye for protein phosphate group staining Pro-Q Diamond, it has been shown that atrophic changes in the gastrocnemius muscle of mice under real microgravity conditions are attended not only by a decrease in the T1 content, but also by a 1.3- and 3.3-fold increase in the degree of phosphorylation of T1 and T2, respectively, Ulanova et al. (2015). Similar changes have been registered in the gastrocnemius and soleus muscles of rats upon the development of alcohol-induced muscle atrophy (Gritsyna et al., 2017). According to certain results, obtained using monoclonal antibodies to phosphoserine pS26, a three-fold increase in the degree of phosphorylation of the PEVK region (located in zone I of the molecule) of titin in the quadriceps muscle of patients with Ehlers-Danlos syndrome was accompanied by a decrease (by ∼20%) in the content of this protein (Ottenheijm et al., 2012). It has been suggested that titin hyperphosphorylation favors an increase in the sensitivity of this protein to proteolysis (Gritsyna et al., 2017).

In the present study, we showed a tendency to higher T2 phosphorylation level after HS. This tendency was accompanied by the enhanced proteolytic degradation of T1. These findings are in agreement with the assumption mentioned above. Administration of L-arginine during unloading caused hypophosphorylation of titin in the rat soleus muscle, that accompanied by a smaller decrease in the level of T1. Collectively, these results suggest that hypophosphorylation of titin, especially T2-part, decreases the susceptibility of this protein to proteolysis. This may be indirectly supported by finding that GSK3β inhibition in mice prevented the phosphorylation and depolymerization of desmin and blocked atrophy upon fasting or denervation. Mass spectrometry analysis identified GSK3-β and calpain-1 bound to desmin and catalyzing its disassembly (Aweida et al., 2018). This study proved that phosphorylation of desmin filaments by GSK3β is a key molecular event required for calpain-1–mediated depolymerization, and the subsequent myofibril destruction.

## Conclusion

In conclusion, we showed that administration of L-arginine, the NO precursor, under gravitational unloading decreased the degree of atrophy changes, increased gene expression of titin and prevented the decrease in levels of T1 in rat soleus muscle. The results can be used to search for approaches to reduce the development of negative changes caused by gravitational unloading in the muscle. Because of limited n-number and high data variability, further research is required to provide evidence of changes in the phosphorylation level of titin and gain insight into how the post-translational modification affect the susceptibility of titin to proteolysis. To find answers to these research questions is the aim of our future studies.

## Ethics Statement

This study was carried out in accordance with the recommendations of Grundy (2015) and the European Convention for the protection of Vertebrate Animals used for Experimental and Scientific purposes (Council of Europe number 123, Strasbourg, 1986). The protocol was approved by the Biomedicine Ethics Committee of the Institute of Biomedical Problems of the Russian Academy of Sciences/Physiology Section of the Russian Bioethics Committee (protocol no. 421 of April 14, 2016). All efforts were made to minimize the animal pain and suffering.

## Data Avaliability Statement

All datasets generated/analyzed for this study are included in the manuscript/Supplementary Files.

## Author Contributions

TN, BS, and IV conceived and designed the work. AU, YG, NS, YL, and SB performed the experiments. AU, BS, and IV analyzed the data and wrote the manuscript. AU and YG prepared the figures. AU, BS, and IV reviewed the manuscript.

## Conflict of Interest

The authors declare that the research was conducted in the absence of any commercial or financial relationships that could be construed as a potential conflict of interest.

## Footnotes

1 https://gygi.med.harvard.edu/phosphomouse/index.php

2 http://cpr1.sund.ku.dk/cgi-bin/PTM.pl

## Abbreviations

C: cage control
HS: 7-day hindlimb suspension
HSL: 7-day hindlimb suspension + L-arginine
NO: nitric oxide
T1: Intact titin 1
T2: proteolytic fragments of T1.
