# Supplementary material for: Effect of L-Arginine on Titin Expression in Rat Soleus Muscle After Hindlimb Unloading
Source: Front Physiol. 2019 Sep 20;10:1221. doi: 10.3389/fphys.2019.01221 (PMC6764413; doi:10.3389/fphys.2019.01221)
Supplement: Supplementary file 1 [file Table_1.docx]

**“Supplementary Tables”**

The statistical analysis of the results obtained was carried out with SigmaPlot 11.0 software (Systat Software, Inc., 2008). Since the distribution of some data samples was not normal (Shapiro-Wilk test), we estimated the significance of differences using nonparametric single-factor dispersion analysis for repeated measurements (Kruskal-Wallis One Way Analysis of Variance on Ranks) with the following pairwise comparison by the Tukey test. The differences were considered statistically significant at р < 0.05.

**Soleus weight, g**

| **Control** | **HS** | **HSL** |
| --- | --- | --- |
| 0.129  0.128  0.118  0.115  0.115 | 0.083  0.072  0.072  0.069  0.064 | 0.087  0.081  0.076  0.073  0.071 |

| Group | N | Missing | Median | 25% | 75% |
| --- | --- | --- | --- | --- | --- |
| **Control** | 5 | 0 | 0.118 | 0.115 | 0.129 |
| **HS** | 5 | 0 | 0.072 | 0.0665 | 0.0775 |
| **HSL** | 5 | 0 | 0.076 | 0.072 | 0.084 |

The differences in the median values among the treatment groups are greater than would be expected by chance; there is a **statistically significant difference (P = 0.006)**

All Pairwise Multiple Comparison Procedures (Tukey Test):

| Comparison | Diff of Ranks | q | P | **P<0.050** |
| --- | --- | --- | --- | --- |
| **Control** vs **HS** | 44.000 | 4.400 | 0.005 | **Yes** |
| **Control** vs **HSL** | 31.000 | 3.100 | 0.073 | No |
| **HSL** vs **HS** | 13.000 | 1.300 | 0.628 | No |

**Body weight, g**

| **Control** | **HS** | **HSL** |
| --- | --- | --- |
| 267.4  259.1  243.6  237.3  234.2 | 221.2  221.5  220.1  210.3  210.2 | 247.4  227.1  221.3  220.3  212.7 |

| Group | N | Missing | Median | 25% | 75% |
| --- | --- | --- | --- | --- | --- |
| **Control** | 5 | 0 | 243.600 | 235.750 | 263.250 |
| **HS** | 5 | 0 | 220.100 | 210.250 | 221.350 |
| **HSL** | 5 | 0 | 221.300 | 216.500 | 237.250 |

The differences in the median values among the treatment groups are greater than would be expected by chance; there is **a statistically significant difference (P = 0.014)**

All Pairwise Multiple Comparison Procedures (Tukey Test):

| Comparison | Diff of Ranks | q | P | P<0.050 |
| --- | --- | --- | --- | --- |
| **Control** vs **HS** | 41.000 | 4.100 | 0.010 | **Yes** |
| **Control** vs **HSL** | 25.000 | 2.500 | 0.181 | No |
| **HSL** vs **HS** | 16.000 | 1.600 | 0.495 | No |

**Soleus weight/body weight ratio, mg/g**

| **Control** | **HS** | **HSL** |
| --- | --- | --- |
| 0.482  0.495  0.483  0.486  0.491 | 0.378  0.326  0.325  0.330  0.303 | 0.353  0.356  0.344  0.335  0.333 |

| Group | N | Missing | Median | 25% | 75% |
| --- | --- | --- | --- | --- | --- |
| **Control** | 5 | 0 | 0.486 | 0.482 | 0.493 |
| **HS** | 5 | 0 | 0.326 | 0.314 | 0.354 |
| **HSL** | 5 | 0 | 0.344 | 0.334 | 0.354 |

The differences in the median values among the treatment groups are greater than would be expected by chance; there is **a statistically significant difference (P = 0.005)**

All Pairwise Multiple Comparison Procedures (Tukey Test):

| Comparison | Diff of Ranks | q | P | P<0.050 |
| --- | --- | --- | --- | --- |
| **Control** vs **HS** | 45.000 | 4.500 | 0.004 | **Yes** |
| **Control** vs **HSL** | 30.000 | 3.000 | 0.086 | No |
| **HSL** vs **HS** | 15.000 | 1.500 | 0.539 | No |

**T1/MyHC**

| **Control** | **HS** | **HSL** |
| --- | --- | --- |
| 0.196  0.204  0.191  0.190  0.201 | 0.151  0.144  0.143  0.160  0.166 | 0.187  0.180  0.171  0.189  0.211 |
| 0.196±0.006 | 0.153±0.010 | 0.188±0.015 |

| Group | N | Missing | Median | 25% | 75% |
| --- | --- | --- | --- | --- | --- |
| **Control** | 5 | 0 | 0.196 | 0.191 | 0.201 |
| **HS** | 5 | 0 | 0.151 | 0.144 | 0.160 |
| **HSL** | 5 | 0 | 0.187 | 0.180 | 0.189 |

The differences in the median values among the treatment groups are greater than would be expected by chance; there is **a statistically significant difference (P = 0.005)**

All Pairwise Multiple Comparison Procedures (Tukey Test):

| Comparison | Diff of Ranks | q | P | P<0.050 |
| --- | --- | --- | --- | --- |
| **Control** vs **HS** | 45.000 | 4.500 | 0.004 | **Yes** |
| **Control** vs **HSL** | 15.000 | 1.500 | 0.539 | No |
| **HSL** vs **HS** | 30.000 | 3.000 | 0.086 | No |

**T2/MyHC**

| **Control** | **HS** | **HSL** |
| --- | --- | --- |
| 0.074  0.072  0.065  0.082  0.069 | 0.092  0.119  0.149  0.136  0.102 | 0.082  0.088  0.097  0.094  0.073 |
| 0.072±0.006 | 0.120±0.023 | 0.087±0.010 |

| Group | N | Missing | Median | 25% | 75% |
| --- | --- | --- | --- | --- | --- |
| **Control** | 5 | 0 | 0.072 | 0.069 | 0.074 |
| **HS** | 5 | 0 | 0.119 | 0.102 | 0.136 |
| **HSL** | 5 | 0 | 0.088 | 0.082 | 0.094 |

The differences in the median values among the treatment groups are greater than would be expected by chance; there is **a statistically significant difference (P = 0.006)**

All Pairwise Multiple Comparison Procedures (Tukey Test):

| Comparison | Diff of Ranks | q | P | P<0.050 |
| --- | --- | --- | --- | --- |
| **Control vs HS** | 45.500 | 4.550 | 0.004 | **Yes** |
| **HSL** vs **HS** | 23.500 | 2.350 | 0.220 | No |
| **Control** vs **HSL** | 22.000 | 2.200 | 0.265 | No |

**T1** **_phosphorylation level_ /T1_protein level_**

| **Control** | **HS** | **HSL** |
| --- | --- | --- |
| 1.383  1.301  1.074  1.265  1.312 | 1.363  1.217  1.542  1.150  1.225 | 1.233  1.179  1.065  1.301  1.212 |
| 1.267±0.116 | 1.299±0.156 | 1.198±0.087 |

| Group | N | Missing | Median | 25% | 75% |
| --- | --- | --- | --- | --- | --- |
| **Control** | 5 | 0 | 1.301 | 1.219 | 1.312 |
| **HS** | 5 | 0 | 1.225 | 1.187 | 1.363 |
| **HSL** | 5 | 0 | 1.212 | 1.064 | 1.233 |

The differences in the median values among the treatment groups are not great enough to exclude the possibility that the difference is due to random sampling variability; there is **not a statistically significant difference (P = 0.359)**

**T2** **_phosphorylation level_ /T2_protein level_**

| **Control** | **HS** | **HSL** |
| --- | --- | --- |
| 1.160  0.960  1.063  0.920  1.077 | 1.140  1.206  1.286  1.051  1.477 | 0.876  1.017  0.760  0.827  0.954 |
| 1.036±0.096 | 1.232±0.162 | 0.887±0.102 |

| Group | N | Missing | Median | 25% | 75% |
| --- | --- | --- | --- | --- | --- |
| **Control** | 5 | 0 | 1.063 | 1.054 | 1.077 |
| **HS** | 5 | 0 | 1.206 | 0.896 | 1.286 |
| **HSL** | 5 | 0 | 0.876 | 0.827 | 0.954 |

The differences in the median values among the treatment groups are greater than would be expected by chance; there **is a statistically significant difference (P = 0.010)**

All Pairwise Multiple Comparison Procedures (Tukey Test):

| Comparison | Diff of Ranks | q | P | P<0.050 |
| --- | --- | --- | --- | --- |
| **HS** vs **HSL** | 43.000 | 4.300 | 0.007 | **Yes** |
| **HS** vs **Control** | 20.000 | 2.000 | 0.333 | No |
| **Control** vs **HSL** | 23.000 | 2.300 | 0.234 | No |

**Titin mRNA levels**

|  | **C, cycles** | **C, %** | **HS, %** | **HSL, %** |
| --- | --- | --- | --- | --- |
|  | 19.7 | 99.5 | 123 | 107 |
|  | 21.6 | 109.1 | 66.7 | 152 |
|  | 18.2 | 91.9 | 53.5 | 174 |
|  | 20.6 | 104 | 71.4 | 132 |
|  | 19 | 95.96 | 120 | 200 |
| Average | 19.8±1.2 | 100.1±6.72 | 86.9±32.26 | 153±36.08 |

| Group | N | Missing | Median | 25% | 75% |
| --- | --- | --- | --- | --- | --- |
| **Control** | 5 | 0 | 99.500 | 95.96 | 104 |
| **HS** | 5 | 0 | 71.400 | 66.7 | 120 |
| **HSL** | 5 | 0 | 152.000 | 132 | 174 |

The differences in the median values among the treatment groups are greater than would be expected by chance; there is **a statistically significant difference (P = 0.025)**

All Pairwise Multiple Comparison Procedures (Tukey Test):

| Comparison | Diff of Ranks | q | P | P<0.050 |
| --- | --- | --- | --- | --- |
| **HSL** vs **HS** | 35.000 | 3.500 | 0.036 | **Yes** |
| **HSL** vs **Control** | 31.000 | 3.100 | 0.073 | No |
| **Control** vs **HS** | 4.000 | 0.400 | 0.957 | No |
