# Supplementary material for: Effect of L-Arginine on Titin Expression in Rat Soleus Muscle After Hindlimb Unloading
Source: Front Physiol. 2019 Sep 20;10:1221. doi: 10.3389/fphys.2019.01221 (PMC6764413; doi:10.3389/fphys.2019.01221)

**“Supplementary Tables”**

DNA fragment of titin gene (*TTN*) is TGTCAGAGCCTCAGAGTATCAGAGTGGTG (sequence data).

<https://blast.ncbi.nlm.nih.gov/Blast.cgi?PROGRAM=blastn&PAGE_TYPE=BlastSearch&LINK_LOC=blasthome>

BLAST program software was used for identification of the PCR product.

This program helps find the mRNA of research product.

The program found that our PCR product is Select seq XM_017592328.1

PREDICTED: Rattus norvegicus titin (Ttn), transcript variant X1, mRNA

58.0 58.0 100% 6e-06 100.00% XM_017592328.1


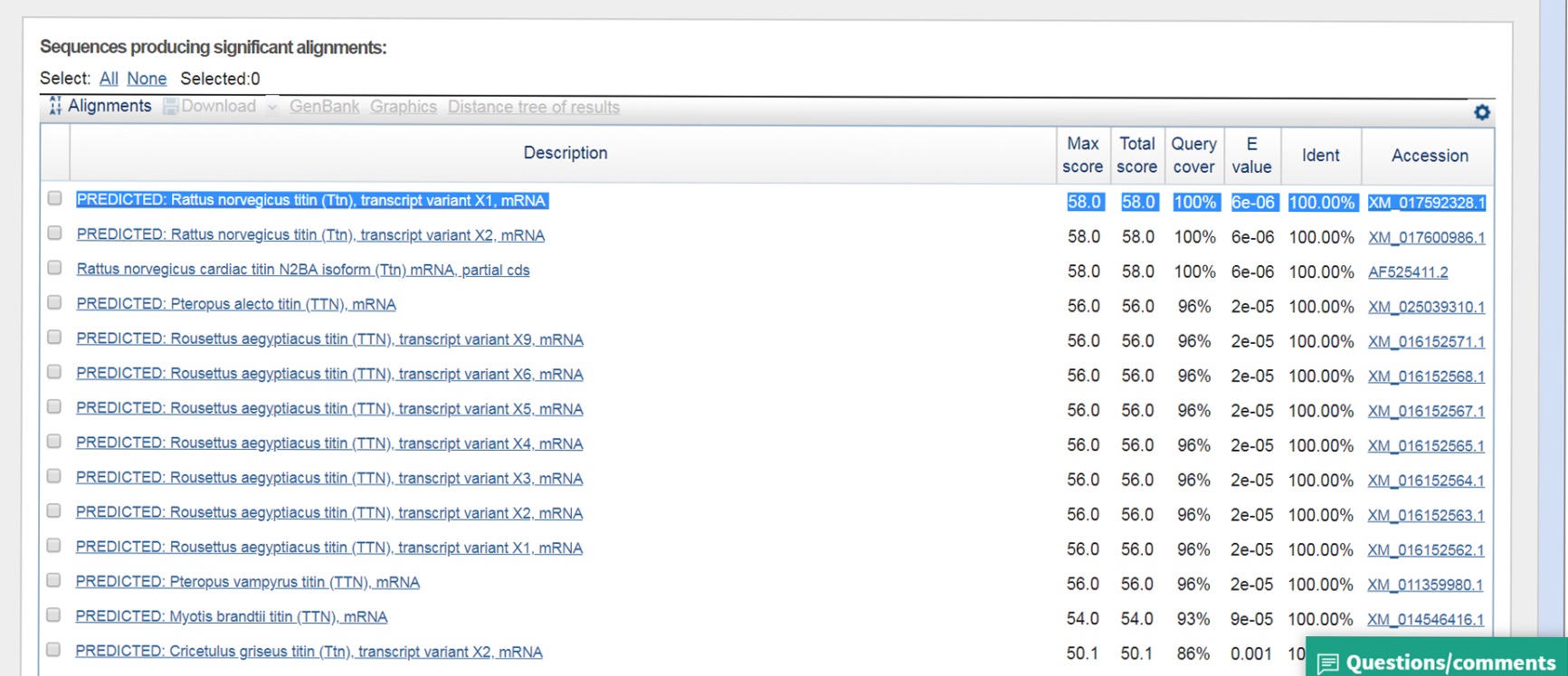

Supplement: Supplementary file 2 [file Table_2.docx]
